# Reduction of the Multipath Propagation Effect in a Hydroacoustic Channel Using Filtration in Cepstrum

**DOI:** 10.3390/s20030751

**Published:** 2020-01-29

**Authors:** Agnieszka Czapiewska, Andrzej Luksza, Ryszard Studanski, Andrzej Zak

**Affiliations:** 1 Faculty of Electronics, Telecommunications and Informatics, Gdansk University of Technology, Narutowicza 11/12, 80-233 Gdańsk, Poland; agnieszka.czapiewska@pg.edu.pl; 2 Faculty of Electrical Engineering, Gdynia Maritime University, Morska 81-87, 81-255 Gdynia, Poland; a.luksza@we.umg.edu.pl (A.L.); r.studanski@we.umg.edu.pl (R.S.); 3 Faculty of Mechanical and Electrical Engineering, Polish Naval Academy, Smidowicz 69, 81-127 Gdynia, Poland

**Keywords:** hydroacoustic channel, sound propagation, multipath propagation, underwater communication, cepstrum

## Abstract

During data transmission in a hydroacoustic channel, one of the problems is the multipath propagation effect, which leads to a decrease in the transmission parameters and sometimes completely prevents it. Therefore, we have attempted to develop a method, which is based on a recorded hydroacoustic signal, that allows us to recreate the original (generated) signal by eliminating the multipath effect. In our method, we use cepstral analysis to eliminate replicas of the generated signal. The method has been tested in simulation and during measurements in a real environment. Additionally, the influence of the method on data transmission in the hydroacoustic channel was tested. The obtained results confirmed the usefulness of the application of the developed method and improved the quality of data transmission by reducing the multipath propagation effect.

## 1. Introduction

Data transmission in the water environment using acoustic waves is applicable in many areas of human activity, including underwater works, marine environment research, underwater sports, and is particularly important in regard to the military. Unfortunately, despite enormous progress in telecommunication, the possibility of using a hydroacoustic channel for communication is very limited, and new solutions are still being sought in this field [1]. This is due to the phenomenon of damping of the mechanical wave in the water, which is the higher, the higher is the frequency; and due to the multipath propagation of the emitted acoustic wave, as well as the non-linear nature of this propagation environment [2,3]. The multipath effect is favored by shallow and/or narrow reservoirs, reservoirs with intense hydrotechnical buildings such as canals or harbors, as well as reservoirs with strong water stratification caused by temperature or salinity. One of such reservoirs is the Baltic Sea basin [3]. In these reservoirs, a significant number of reflections and a long time of the hydroacoustic channel memory play a significant role [4]. In data transmission systems, the phenomenon of the multipath causes the inter-symbol interference disturbance, including even hundreds of symbols, which significantly hampers communication [1].

According to the above, the main purpose of the study is to develop a method, which is based on a recorded hydroacoustic signal, that allows us to recreate the original (generated) signal by reducing the multipath effect.

This article is dedicated to research carried out in the area of hydroacoustics and telecommunication. It must be noted that methods of reduction of the multipath propagation effect (echo reduction, echo cancellation, and echo suppression) are commonly used in telephony [5,6,7]. There are some reports of using this kind of signal processing in radiocommunication. In such cases, adaptive filtration [8,9], blind separation [10] or convolution methods with the inverse impulse response of the transmission channel [11] are most often used. These methods suffer from one serious disadvantage, i.e., the transmission channel must be stationary in a relatively long time. In the case of underwater communication, this condition can rarely be met. Other described in literature interesting methods of echo cancellation are the combination of particle filtering and evolutionary strategies [12], novel frequency-domain second-order Volterra filter based on soft decision [13], and Kalman filters (used with stereophones) [14]. Cepstrum analysis is widely used in signal processing in many areas. For example to glottal flow estimation [15], identification of damage in civil engineering structures [16], detection of voice disorders [17], detection of emotions [18], estimating the heart rate from arrays of fiber Bragg grating (FBG) sensors [19], heart rate estimation (improve clarity of data from photoplethysmography) [20], and improve the detection quality of micro changes in biological structures [21]. There are also few publications in the literature regarding the reduction of the multipath effect in the hydroacoustic channel during data transmission, such as [22], where authors are using pulse position modulation spread spectrum underwater acoustic communication system using the N-H sequence.

The article is organized as follows: Section 2 describes in details the method of reduction of the multipath propagation effect and adopted assessments indicators, Section 3 presents issues related to the tuning method’s parameters, Section 4 presents the result of the research obtained during simulation and then during measurements conducted in real environment conditions, including results of influence of the presented method on data transmission in the hydroacoustic channel, as well as discussion of the obtained results of the research, and Section 5 is a summary.

## 2. Method Description

Sound propagation in water is a complex problem. It depends on many things, for example: the physico-chemical properties of water (including layering of water), type of the bottom, occurrence of natural or artificial obstacles (including hydrotechnical buildings), distance between transmitter and receiver (including its relative position), frequency of transmitting signals, etc. [3]. In simplified terms, we can say that in free space propagation, sound pressure is inversely proportional to the distance between the transmitter and receiver. Sound reflects from the boundary of a water region (bottoms, water’s surface, immersed objects, or hydrotechnical buildings, i.e., in harbors). Every reflection attenuates the sound. The value of attenuation depends on the signal frequency and the material from which the object is made, from which the sound is reflected. We can model them by a single coefficient, $\alpha \in \left[0,1\right],$ which means a decrease in the amplitude of the signal after reflection. Water region, from the point of view of multipath sound propagation, can be treated as a linear filter, which acts by convolution. So, the received sound is the emitted sound convolved with a hydroacoustic channel impulse response, which can be written as follows:

(1) $$x\left(n\right)=s\left(n\right)*h\left(n,p\right)$$

where: $x\left(n\right)$—received signal, $s\left(n\right)$—transmitted signal, $h\left(n,p\right)$—impulse response of water region measured in location p, and n—discrete time (sample). 

The transmitted signal $s\left(n\right)$ is a sinusoidal one with a specified frequency $f_{c}$ modulated by the pseudo-random sequence, thus we can write as follow:

(2) $$s\left(n\right)=z\left(n\right)\sin\left(2\pi f_{c}n\right)$$

where: $z\left(n\right)$—represents a pseudo-random sequence, and $f_{c}$—carrier frequency.

Estimation of hydroacoustic channel impulse response can be determined using a pseudo-random binary sequence [23,24]. The estimate of the channel’s impulse response is determined using a module of the cross-correlation function between the pseudo-random binary sequence and the received signal brought to the baseband [25]. The cross-correlation is a scalar product of two signals in the offset function of one of them, which can be calculated according to the formula [26]:

(3) $$R_{zy}\left(k\right)=\overset{N-1-\left|k\right|}{\underset{n=0}{\sum }}z\left(n\right)y^{*}\left(n-k\right)$$

where $y\left(n\right)$ is the received signal brought to the baseband. 

Based on (3) the estimation of the impulse response of the hydroacoustic channel can be determined calculating the module as follows [26]:

(4) $$h\left(k\right)=\sqrt{{\left(\overset{N-1-\left|k\right|}{\underset{n=0}{\sum }}z\left(n\right)y_{s}^{*}\left(n-k\right)\right)}^{2}+{\left(\overset{N-1-\left|k\right|}{\underset{n=0}{\sum }}z\left(n\right)y_{c}^{*}\left(n-k\right)\right)}^{2}}$$

where: $y_{s}\left(n\right)=x\left(n\right)\sin\left(2\pi f_{c}n\right)\;\mathrm{and}\;y_{c}\left(n\right)=x\left(n\right)\cos\left(2\pi f_{c}n\right)$.

**Figure 1:**
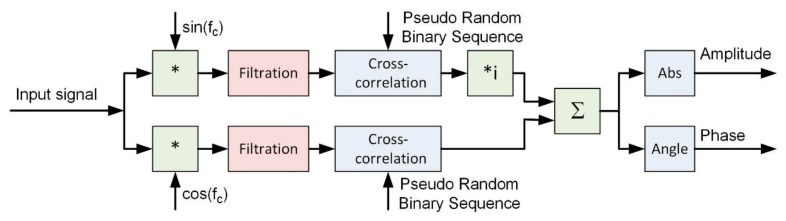
The block diagram of signal processing during the determination of the estimation of the hydroacoustic channel’s impulse response.

Figure 1 shows block diagram of signal processing during the determination of the estimation of the hydroacoustic channel’s impulse response.

In the impulse response of the hydroacoustic channel, there are maxima, which correspond to the next replicas of the transmitted signal, which are reaching a receiver as a result of the multipath propagation.

According to the above, we could assume that as a result of the multipath effect, in the received signal, the transmitted signal and its replicas created by reflections of the signal transmitted from obstacles (including bottom, surface), have been added to each other. Delay of replicas depends on a length of the path of propagation and amplitude depends on the material of an obstacle. Thus we can write formula as follows [27]:

(5) $$x\left(n\right)=s\left(n\right)+\sum_{i=1}^{M}\alpha_{i}s\left(n-n_{i}\right)+w\left(n\right)$$

where: $x\left(n\right)$—received signal, $s\left(n\right)$—transmitted signal, $w\left(n\right)$—ambient noise (e.g., white noise), n—discrete time (sample), $\alpha_{i}$—the amplitude factor of the i-th replica, $n_{i}$—delay of the i-th replica resulting from the multipath propagation, and M—the number of significant replicas (echoes) of the transmitted signal.

The basis of the adopted method of multipath elimination is the observation showing that as a result of the cepstral signal analysis, there are maxima corresponding to the replicas of original signal delays. The complex cepstrum, or the inverse Fourier transform, of the spectrum of the signal expressed in a logarithmic scale, is defined as follows [28,29]:

(6) $$c\left(n\right)=C\left(x\left(n\right)\right)=F^{-1}\left(ln\left(F\left(x\left(n\right)\right)\right)\right)$$

where: F—Fourier transform and $F^{-1}$—inverse Fourier transform.

Please note, that in the article, for simplicity, we will use the name cepstrum meaning complex cepstrum. The cepstrum contains components related to the replicas-free signal, as well as the components resulting from the presence of replicas [30,31]. It should be noted that the cepstrum is symmetrical in relation to half of the length of the analyzed signal. Therefore, components derived from replicas appear on both the left and the right side of the cepstrum. Additionally, if there are several replicas of the original signal (which is natural in real conditions), not only the components resulting from the delays of these replicas appear, but also the components resulting from the differences and sums of delays in arriving at individual replicas. The research shows that in the case of elimination of the multipath effect, first of all, only the components on the left side of the cepstrum should be filtrated (see Section 3.1); it also shows that not only the components resulting from delays in reaching individual replicas, but also those resulting from the combination of subtracting and adding up these delays, should be filtrated. The application of these two principles in the conducted research led to the best results. In the real situation, the delays in arriving at individual replicas are not known in advance, therefore the selection of components for filtration takes place by searching local maximas in cepstrum in selected range, which we named the depth of filtration (see Section 3.2). The research shows that filtration of the selected components brings a better effect than filtration of all components in a given cepstrum. In our method, we proposed to filtrate only the given number (see Section 3.3) of components around locally maximum values. 

The process of the cepstrum filtration can be expressed as follows:

(7) $$c\left(m+k\right)=c\left(m+k\right)g\left(k\right),\;k=-l\ldots l$$

where: m—index of the selected component for filtration, l—half the window length, and $g\left(k\right)$—filtration window (in this case $g\left(k\right)=0$).

The cepstrum function is a reversible function. Knowing the cepstrum, it is possible to reproduce the original signal based on the transformation [28,29]:

(8) $$x\left(n\right)=C^{-1}\left(c\left(n\right)\right)=F^{-1}\left(exp\left(F\left(C\left(x\left(n\right)\right)\right)\right)\right)$$

**Figure 2:**
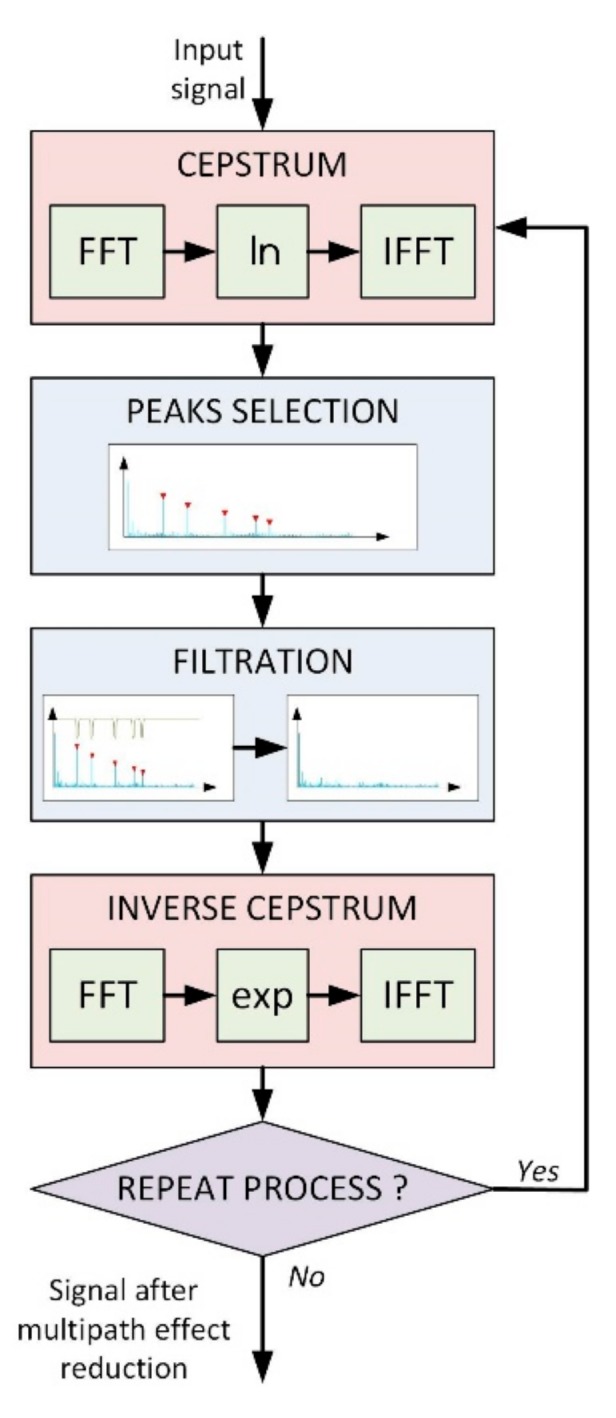
The block diagram of signal processing during the multipath effect reduction.

Figure 2 presents a block diagram of the system performing the operation of multipath effect elimination from the input signal.

Our research confirmed that the whole process of the multipath effect reduction should be repeated several times to get the best result (see Section 3.4).

Assessment of the effectiveness of the adopted method will be carried out on the basis of the comparison of channel impulse response estimates determined for the recorded signal before, and after multipath effect elimination. The most important will be the change of levels of the significant replicas identified in channel impulse response.

As second assessment we will use statistical parameters, which characterize constellation during digital data transmission. During research we will use Binary Phase-Shift Keying (BPSK) modulation (sometimes called Phase Reversal Keying PRK or Two Phase Shift Keying 2PSK), which is the simplest form of phase shift keying [32]. We will use two phases 0 and 180 degrees. During demodulation, each symbol can be represented by a complex number, and the constellation diagram can be regarded as a complex plain with the horizontal, real axis representing the in-phase (I component) carrier (sine wave), and vertical imaginary axis representing the quadrature (Q component) carrier (cosine wave). An ideal constellation diagram for BPSK will show two positions of the point representing each symbol. However, because of noise, distortion, multipath propagation, carrier frequency change, etc. during passing through communication channel, the values of amplitude and phase after demodulation may differ from the correct value for each symbol. Due to that, the point on the constellation diagram, representing received symbols, will be offset from the correct position of symbol. If the aforementioned phenomenon causes a significant dispersion, it can lead to a situation in which the point representing the symbol will be in the region represented by another symbol. In this situation, the demodulator will misidentify that symbol, which results in an error [33]. One of the measures of dispersion is variance. We will determine this value only for the I axis, because of using BPSK modulation, before and after echoes reduction, which should show the influence of the above presented method on the possibility of correcting data reception. To calculate these parameters, we will use the following formulas:

(9) $$\sigma_{I}^{2}=\frac{1}{m}\overset{M-1}{\underset{m=0}{\sum }}{\left(y_{Ic}\left(m\right)-\mu_{I}\right)}^{2}$$

where:

(10) $$\mu_{I}=\frac{1}{m}\overset{M-1}{\underset{m=0}{\sum }}y_{Ic}\left(m\right)$$

(11) $$y_{Ic}\left(m\right)=\left|\overset{L-1}{\underset{l=0}{\sum }}y_{c}\left(m+l\right)\right|$$

where: L—length of a single symbol in samples. 

In order to be able to compare the results before and after the application of the described method of reduction of the multipath propagation effect, the constellation obtained after filtration is normalized with respect to the coefficient resulting from the ratio of the mean value before filtration, to the mean value after filtration. For such a modified constellation, the variance after the reduction of the multipath effect was calculated. Heaving values of variations before and after filtration we could determine the I quality improvement factor using the following equation:

(12) $$v_{I}=\frac{\sigma_{I_{before}}^{2}}{\sigma_{I_{after}}^{2}}$$

where: $\sigma_{I_{before}}^{2}$ and $\sigma_{I_{after}}^{2}$—variance before and after reduction of multipath propagation effect, respectively.

The I quality improvement factor will be higher than one, if the quality of transmission has improved. In the case of a value equal to one, there will be no change, and a value below one will mean a loss of transmission quality.

## 3. Tuning Method’s Parameters

In order to obtain optimal operating conditions for the described method, simulation tests were carried out to determine the impact of selected parameters on the quality of receiving BPSK signal. The influence of the following parameters on the method of operation, and the quality of the obtained results were tested:

- the depth of filtration in the cepstrum;
- width of the filtration window;
- number of iterations during cepstrum filtration.

As a depth of filtration in the cepstrum we will understand the value of the frequency to which we will search peaks in the cepstrum. All peaks in this range will be filtered.

The tests were carried out taking into account various replica delays in relation to the original signal. More over researches were performed for various values of sampling frequencies, carrier frequencies, and modulated signal bandwidths. The obtained results were similar.

Presented bellow results were obtained for the following signal parameters:

- BPSK modulated signal;
- length of the spreading sequence 63;
- number of bits transmitted in frame 280;
- preamble length (pilot): 14 bits with value 1; preamble bits are also spread.
- number of chips spreading a single bit: 9;
- modulated signal bandwidth: 50 kHz;
- sampling frequency: 500 kHz;
- carrier frequency: 100 kHz;
- SNR = 20 dB;
- amplitude of the first replica: 0.95.

The quality of the reception was determined in accordance with (12). 

The method requires determining the value of individual parameters so as to obtain the best result of its operation. Since the problem is too complex to predict the result using an analytical approach, it was decided to carry out simulation tests. For this purpose, it was assumed that the I quality improvement factor will be an endogenous (forecast) variable. Its value will be determined according to (12) for each set of individual simulation parameters separately. Individual simulation parameters, i.e., the depth of filtration in the cepstrum, the width of the filtration window, the number of iterations will be exogenous (predictive) variables. All exogenous variables will have a uniform distribution. We are interested in the answer to the question for which set of parameters values we will get the best result of quality improvement (the maximum value). It should allow us to determine the optimal set of parameter values, which will lead us to maximally increase transmission quality improvement. After conducting tests in which we simulated 500,000 transmissions, the obtained result allowed us to conclude that the optimal parameter values, because of maximizing the I quality improvement factor, were equal to, for the depth of filtration in cepstrum, 12 ms, the width of the filtration window, 9 samples, and the number of iterations, 6.

The impact of the change of values of selected parameters on the methods performance was researched. Results of this research are presented in the following subsections. In these studies the average value of the I quality improvement factor as well as the number of cases where the data transmission quality improve in relation to all tests were determined as to judge the method performance.

### 3.1. Selection of the Filtration Side in the Cepstrum

**Figure 3:**
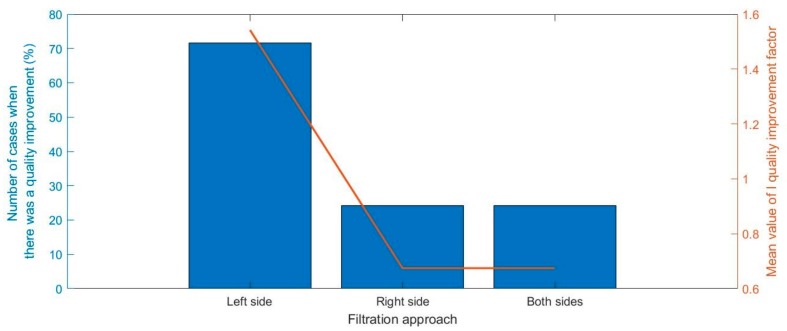
The impact of selecting the side of filtration in the cepstrum to the number of cases when there was a quality improvement and mean value of the I quality improvement factor.

**Figure 4:**
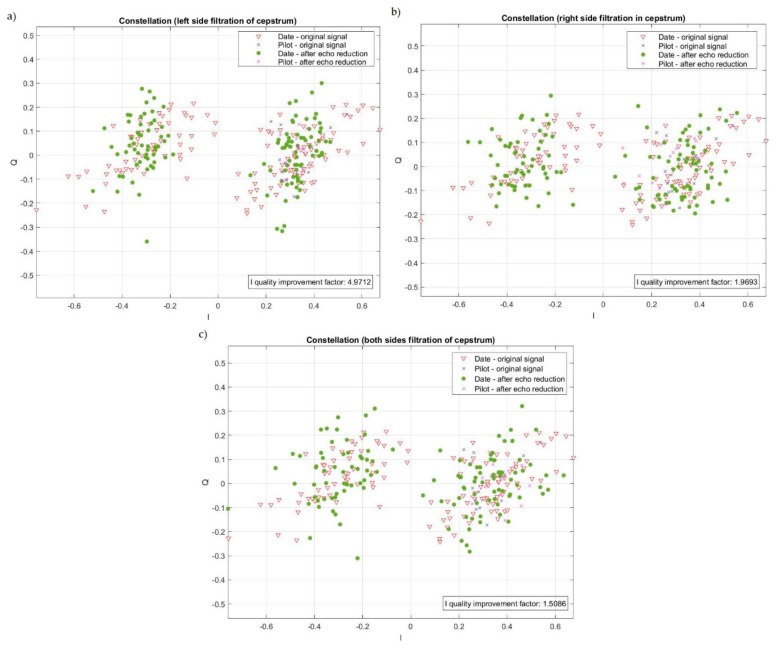
An example of the impact of the method performance when filtration was realized on the (**a**) left side only, (**b**) right side only, and (**c**) both sides.

Since components derived from replicas appeared on both the left and the right side of the cepstrum there was a problem of the selection side of filtration. There is a possibility of filtering components coming from replicas only on the left side, only on the right side, or on both sides. After conducting tests in which we simulated 50,000 transmissions, for randomly selected impulse responses, i.e., time delays of replicas and their amplitudes, the obtain results allowed us to conclude that cepstrum should be filtered only on the left side. As it is shown in Figure 3, filtration of cepstrum only on the left side gave the best results, which means the maximum of the I quality improvement factor and maximum number of cases when there was a quality improvement. It must be noted that the value of the I quality improvement factor below 1 means a deterioration of quality. Figure 4 shows an example of the impact of selecting the side of filtration in the cepstrum on the method performance.

In this specific case during filtration of the left side the value of the I quality improvement factor was 4.9712. For the other case the value of the I quality improvement factor was 1.9693 and 1.5086 for filtration in cepstrum on the right side and both sides respectively. As the test results show, the best result was obtained by filtering the components only on the left side of the cepstrum and this approach will be applied in the next researches.

### 3.2. Depth of Filtration in the Cepstrum

**Figure 5:**
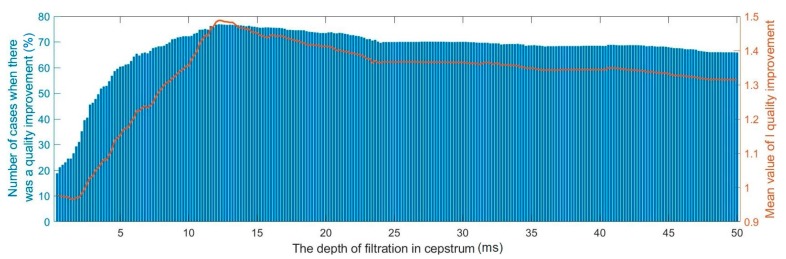
Influence of the depth of filtration in the cepstrum on the I quality improvement factor.

In practical solutions, we do not know the number of replicas that will occur in the received signal. To a large extent, it depends on the propagation conditions in the given area, and in particular, on the obstacles that occur. Therefore, it should be specified how many local maxima in the cepstrum have to be filtered. Too small of a value will not allow it to properly reduce the occurring multipath effect, and too large will cause a deterioration of the signal noise ratio. To indicate the peaks for filtration, we assumed that they would be sought in a specific range, starting from the beginning of the cepstrum. This will allow us to become independent of the number of received replicas. It was assumed that the width of the filtration window would be 9. The test results are shown in Figure 5.

It can be seen that for depth of filtration in the cepstrum equal to 12 ms, the average I quality improvement factor took the highest value. In this point, we could observe also the highest number of cases of quality improvement. Accordingly, in further studies we assumed that the depth of filtration in the cepstrum should be equal to 12 ms.

### 3.3. Width of the Filtration Window

**Figure 6:**
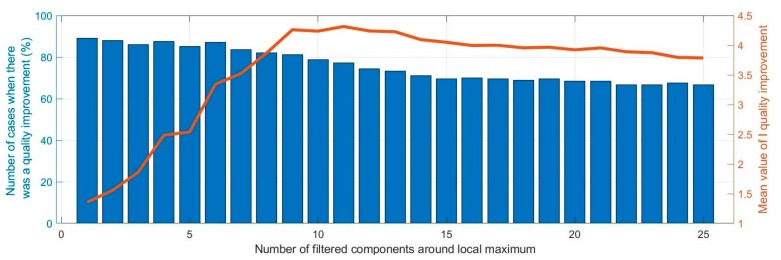
Impact of the width of the filtration window on the I quality improvement factor.

The width of the filtering window determines the number of filtered components around the local maxima. The obtained results are shown in Figure 6.

The analysis of the results shows that the narrower the filtration window, the more the proposed method had improved the quality of reception in more cases. However, it can also be seen that up to a window width of 9, the value of the quality improvement parameter increased. For a larger number of zeroed components, the value of this parameter began to decrease slightly. The above considerations confirmed the results obtained in research described at the beginning of Section 3 that the filtration window used to filter local maxima should be 9 to achieve the best performance of the proposed method.

### 3.4. Number of Iteration 

**Figure 7:**
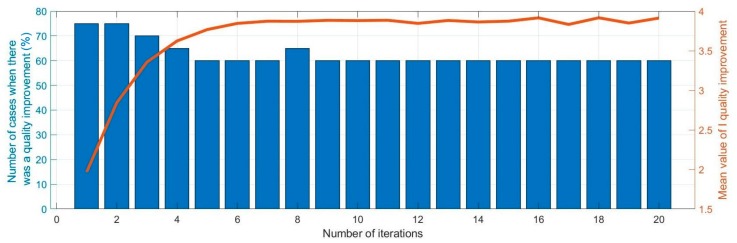
Impact of the number of iterations of filtration on the I quality improvement factor.

Another parameter of the proposed method that can affect the quality of reception is the number of filtration replicas. It has been assumed that all transformations are repeated, it means the entire procedure. Repetition of the entire process is necessary because it has been assumed that the cepstral components are filtered only on the left side of the cepstrum (see Section 3.1). Therefore, on the right side there remain components that during the inverse cepstral transform are revealed in the signal in the form of replicas of the original signal but with a lower amplitude. The tests showed that repeated elimination of replicas allows for a significant reduction of their amplitude. The influence of the number of iterations of filtration on the quality of reception was examined. The results are presented in Figure 7.

It can be seen that for the 6th iteration, the obtained I quality improvement factor reaches a value that does not change with the increase in the number of iterations. The number of quality improvement cases is also stable for the number of iterations greater than six. Unfortunately, the number of these cases is smaller for six iterations than for one iteration. However, it was recognized that it is more important that the procedures work stably, and the effects of quality improvement are more pronounced (a higher value of the relative quality improvement parameter). Therefore, it is recommended to use six iterations of filtration.

## 4. Results of the Research

### 4.1. Simulation Experiment

Simulation experiments were carried out using the Matlab environment. Generated digital signal, which will be treated as a received signal in real conditions, consisted of the four replicas of the basic signal. The first replica is the transmitted signal reaching the receiver with the shortest path. Its amplitude was set to 1. Other replicas resulted from the effect of multipath propagation. Its amplitudes were set to: 0.7, 0.6, and 0.31 respectively. The transmitted signal was a sinusoidal signal modulated with a pseudorandom binary sequence using BPSK modulation. During the simulation, the following parameter values were set: sampling frequency: 500 ksamples/s, carrier frequency of the signal: 12 kHz, number of samples per symbol: 17, and length of the PRBS: 2047 bits. 

**Figure 8:**
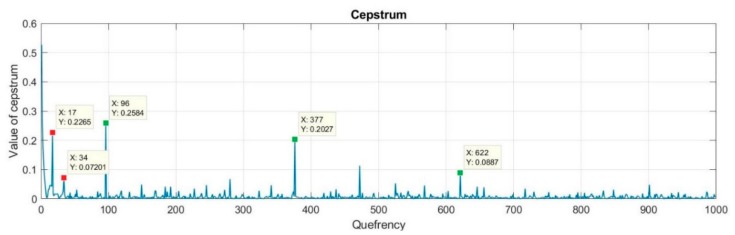
Part of the cepstrum of the input signal with marked components resulting from replicas (green color) and component results from the modulation rate (red color).

Figure 8 shows a fragment of the input signal cepstrum together with the marked components resulting from individual replicas (green color). As it is shown, the second third and fourth replicas were delayed relative to the first replica by 0.194 (ms; 97 samples), 0.754 (ms; 377 samples), and 1.244 (ms; 622 samples) respectively. In this figure, there were also marked components, which resulted from the modulation rate and its first harmonic (red color).

**Figure 9:**
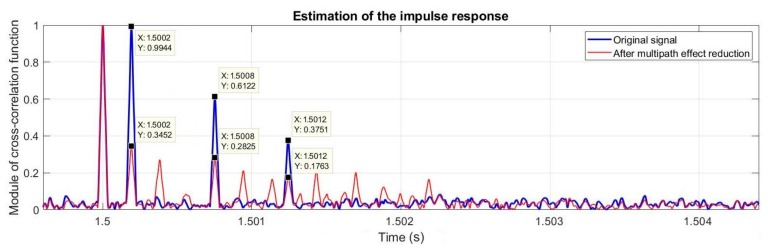
The estimation of the impulse response of the original signal and after elimination of the multipath effect.

Figure 9 shows the estimation of an impulse response of the input signal and the signal after the elimination of the multipath effect processed according to the above described procedure for optimal values of method parameters. The second, third, and fourth replicas are clearly seen in this figure.

It can be clearly seen in Figure 9, that the second, third, and fourth replicas, which were echoes of the transmitted signal, were reduced in the input signal. At the same time, as a result of processing, according to the presented algorithm, additional components appear in the estimation of the impulse response with a higher level than in the original signal. This may be perceived as an ineffective phenomenon of processing, however, the level of these components is much lower than of the elimination components.

**Figure 10:**
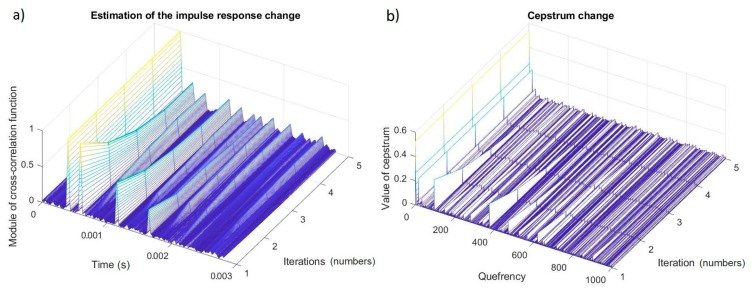
The change of (**a**) the cepstrum and (**b**) estimation of the impulse response of the original signal during the process of elimination of the multipath effect.

The process of a reduction of the multipath effect in the field of estimation of the impulse response and cepstrum changes in subsequent stages (iterations) is presented in Figure 10.

These drawings clearly show the run of the process in which components resulting from the multipath propagation effect were reduced and, at the same time; the unfavorable effect of processing consisting of an increase in the level of some components was visible.

### 4.2. Real Conditions Experiment

Experiments using physical signals were carried out in the real environment. Research was carried out on inland waters on the lake Kosobudno in the village Czernica. Transmitting and receiving hydrophone were lowered from the floating jetty at a distance of about 12 m from shore to a depth of 2 m. The depth of the water area in this point was 3.5 m. The distance between the hydrophones was changed in the range from 4 to 36 m. The bottom in the test area was sandy without underwater vegetation. The immersion depth of the floating jetty was about 0.4 m.

**Figure 11:**
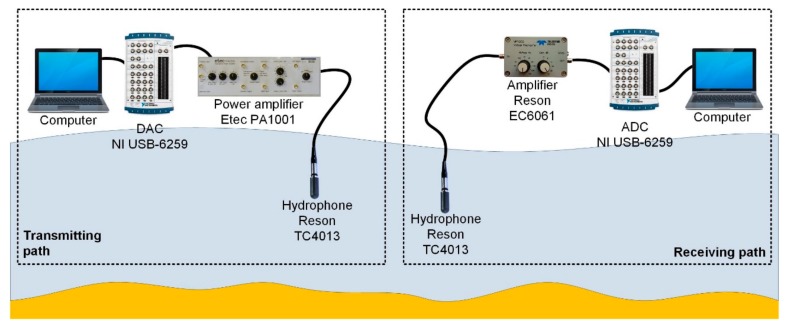
The block diagram of the measurement system.

During tests we used a measuring system, which included the transmission path and the receiving path as it is shown in Figure 11.

The transmitting path consisted of a laptop with NI SignalExpress software, a NI USB-6259 multifunction I/O device, an Etec PA1001 power amplifier, and Reson TC4013 hydrophone. The receiving path consisted of a Reson TC4013 hydrophone, Reson EC6061 amplifier, NI-9222 voltage input module, and laptop with NI SignalExpress software.

In the conducted tests, a sinusoidal signal with BPSK modulation was used as the extortion. The signal with a different carrier frequency (in range of 10–150 kHz) was modulated at a speed of 20 ksymbols/s with a PRBS of length 2047 bits. The sampling frequency was 500 kHz.

**Figure 12:**
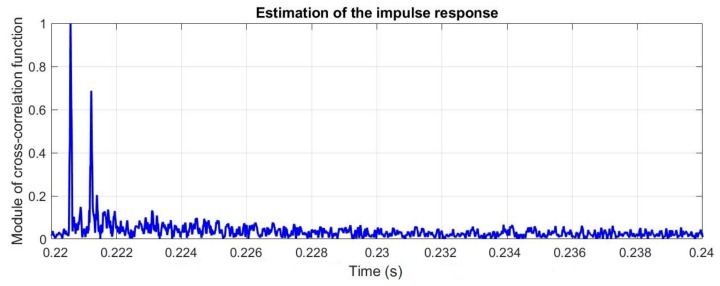
The estimation of the impulse response for the measured signal for the real environment experiment.

Figure 12 presents the estimations of impulse responses obtained for registered (original) signals.

As it is shown, and what was expected, there was only one significant echo produced from the bottom of the floating jetty.

**Figure 13:**
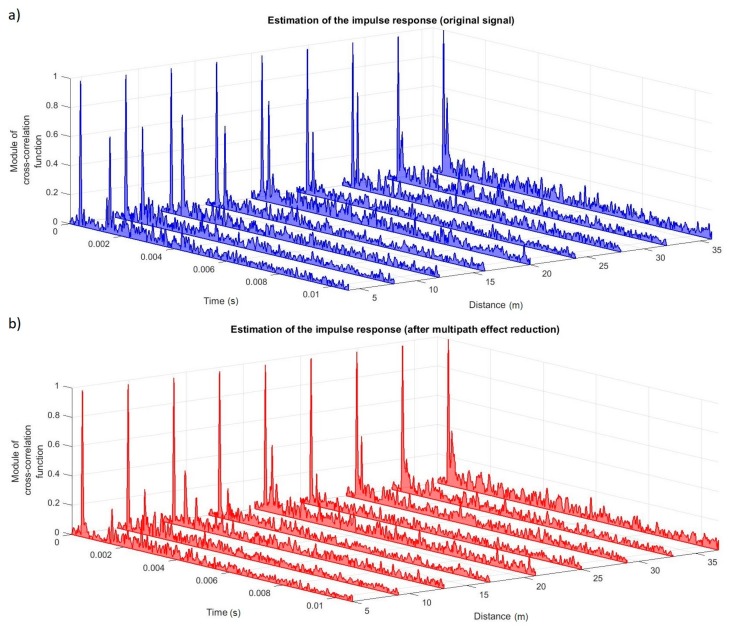
The changes of the impulse responses due to change of distance between the transmitter and receiver, (**a**) before and (**b**) after the multipath effect reduction for the real environment experiment.

In order to investigate the impact of the method of reducing the echo of a transmitted signal depending on the distance between hydrophones, estimates of the impulse response were determined for individual distances before and after the multipath effect reduction. The results are shown in Figure 13. In all cases, an echo reduction about two times compared to the original signal was obtained.

**Figure 14:**
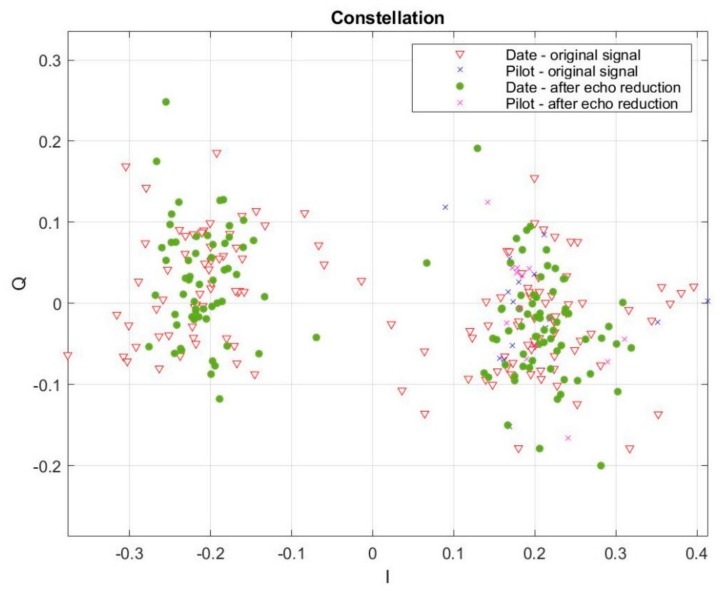
Constellation during data transmission before and after reduction of the multipath effect for a real environment experiment (carrier frequency 20 kHz, bandwidth 30 kHz and distance between hydrophones 4 m).

**Figure 15:**
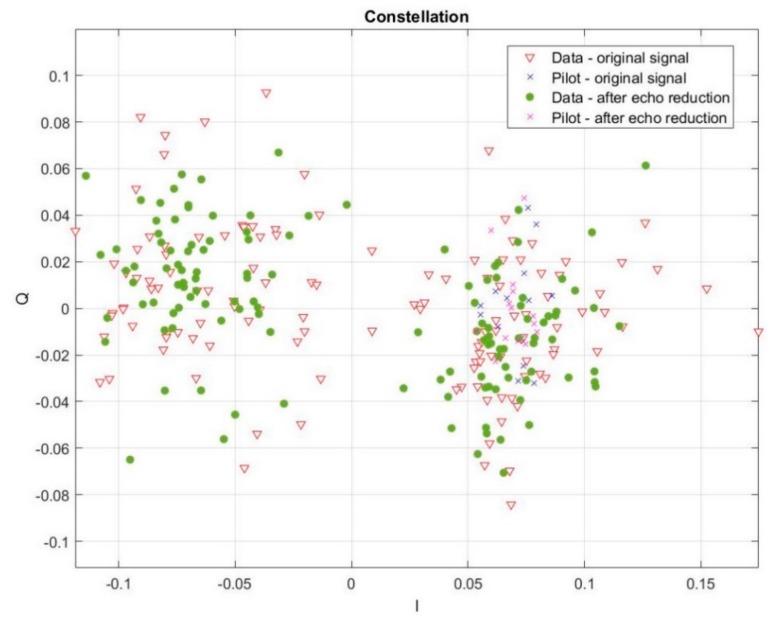
Constellation during data transmission before and after reduction of the multipath effect for a real environment experiment (carrier frequency 100 kHz, bandwidth 120 kHz and distance between hydrophones 36 m).

During real conditions measurements, we also researched the influence of our method of the multipath effect reduction on data transmission in the hydroacoustic channel. Using the same measurement system, we transmitted information organized as follows: first 14 bits were logical ones (synchronization bits—pilots) and the next 140 bits were data bits. Data were transmitted using bandwidth from 10 to 120 kHz. A receiving signal, in the purpose of the multipath effect reduction, was processed accordingly to the above presented method. The influence of our method on the quality of data transmission was rated based on the constellation, especially mean and variation values for part I and Q. Figure 14 and Figure 15 present an example of the constellation for the signal transmitted at a carrier frequency equal to 20 kHz bandwidth 30 kHz, and carrier frequency 100 kHz bandwidth 120 kHz.

In the first case, (Figure 14) the value of the I quality improvement factor was equal to 2.8446. In the second case, (Figure 15) the value of I quality improvement factor was equal to 2.1605. As it is shown in the figures above, the value of variation for part I was lower after the multipath effect elimination. It means that the spread of data is smaller, and at the same time, the possibility of distinguishing the logical state of the data is higher. It causes the quality of transmission to become better. Moreover, the distance of bits, formed a decision line (for BPSK it was a horizontal line on I = 0 position) on the constellation, after the multipath effect reduction became greater, which clearly indicates an improvement in the possibility of separating individual logic states of transmitted data.

**Figure 16:**
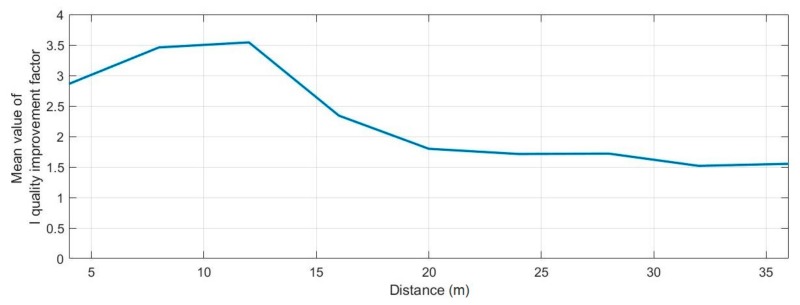
The mean value of the I quality improvement factor during data transmission due to the change of distance between the transmitter and receiver for a real environment experiment.

The measurements were analyzed to determine the mean value of the I quality improvement factor for data transmission in real conditions depending on the distance between hydrophones. The mean value was determined for 30 transmissions over a given distance. The obtained result is presented in Figure 16.

The research shows that the value of the I quality improvement factor decreased as the distance increased. The reason might be a decrease in the time interval between the original signal (which arrived via the direct route) and the echoes from the reflections.

## 5. Conclusions

The main problem in the transmission of digital data in the marine environment in shallow water areas, or in areas with high hydrotechnical building, is the occurrence of the multipath effect. It negatively affects the quality of the transmitted data.

The main purpose of the study was to develop a method, which is based on a recorded hydroacoustic signal, that allows us to recreate the original (generated) signal by reducing the multipath effect. Such an action should ensure an increase in the quality of digital data transmission, and more precisely, increase the likelihood of the correct distinction between the individual logical states of transmitted data. In order to solve this task, the filtering method used in the cepstrum of the received signal was developed. By eliminating certain cepstral components and reconstructing signal, the multipath effect was reduced. In order to determine the quality improvement obtained by signal processing, a quality improvement factor was proposed. This coefficient was based on the variance of the constellations for individual bits. The effectiveness of the developed method depends on several parameters. Therefore, in order to achieve optimal improvement of the quality of transmission, the research was carried out, giving an answer about the values of individual parameters. The correctness of the method’s operation has been confirmed in simulation tests and tests in real conditions.

Simulation tests show the possibility of reducing multipath effects by filtering the cepstrum of received signals. Experiments in real conditions confirmed the correctness of the adopted solutions. During the research carried out for various carrier frequencies and bandwidth, as well as relative positioning of the sensors, the transmission quality was improved by up to five times. This value should allow for an uninterrupted transmission in difficult propagation conditions, i.e., in the case of a multipath effect. The conducted research indicates that the developed method can be successfully used as an input stage in the receiver path.

In future studies, the method should be evaluated in the case of even more difficult propagation conditions, i.e., when there are more replicas of the transmitted signal. It is also important to consider the possibilities of the method implementation including hardware implementation for real-time signal processing.
